# Treatment Algorithms for Inflammatory Myopathies in Adults: from Guidelines to Clinical Practice

**DOI:** 10.1007/s40674-025-00239-5

**Published:** 2025-12-29

**Authors:** Albert Selva-O’Callaghan, Ernesto Trallero-Araguás, Albert Gil-Vila, Ana Matas-Garcia, Clara Edo, Jose Milisenda, Iago Pinal-Fernández

**Affiliations:** 1 Systemic Autoimmune Diseases Unit, Internal Medicine Department, Vall d’Hebron General Hospital, Universitat Autònoma de Barcelona, Barcelona, Spain; 2 Rheumatology Department, Vall d’Hebron Hospital, Barcelona, Spain; 3 Muscle Research Unit, Internal Medicine Service, Hospital Clinic, Barcelona University, CIBERER and IDIBAPS, Barcelona, Spain; 4 Muscle Disease Unit, National Institute of Arthritis and Musculoskeletal and Skin Diseases, National Institutes of Health, Bethesda, MD, USA; 5 Department of Neurology, Johns Hopkins University School of Medicine, Baltimore, MD, USA

**Keywords:** Inflammatory myopathy, Myositis, Dermatomyositis, Antisynthetase syndrome, Immune-mediated necrotizing myopathy, Myositis guidelines, Myositis treating algorithm, Myositis clinical practice

## Abstract

**Purpose of Review:**

Inflammatory myopathies are a heterogeneous group of systemic autoimmune disorders with highly variable clinical presentations. The limited number of clinical trials has hindered the development of strong evidence-based management strategies. Therefore, there is a pressing need for consensus-driven treatment guidelines tailored to the various clinical and immunological phenotypes. This review combines existing published guidelines with the authors’ clinical experience to provide a comprehensive, pragmatic approach to management.

**Recent Findings:**

A comparative review of existing treatment guidelines was recently published, revealing significant heterogeneity in therapeutic approaches and recommendations. This observation is consistent with our own experience during a multidisciplinary meeting, primarily involving internal medicine specialists and rheumatologists. Furthermore, recent clinical trials and observational studies investigating novel therapies offer promising prospects that could influence future clinical decision-making.

**Summary:**

Although establishing a unified therapeutic algorithm remains challenging, this review aims to translate current knowledge into clinical practice by integrating existing guidelines, our own clinical experience, and the most recent evidence published on the topic.

## Introduction

Inflammatory myopathies (IM), collectively referred to as myositis, are considered systemic disorders of autoimmune origin. Although skeletal muscle is the primary organ affected, other systems such as the skin, joints, and lungs are frequently involved. More recently, involvement of the gastrointestinal tract and heart has also been recognized as part of the disease spectrum. This is an heterogenous group of diseases that has recently been better defined and classified, thanks to collaborative efforts by scientific organizations and the identification of novel autoantibodies [1, 2]. However, despite these advances, the wide variability in clinical manifestations continues to pose significant challenges in establishing standardized therapeutic algorithms applicable in clinical practice. Moreover, robust scientific evidence supporting the most effective treatment strategies for these complex patients remains limited [3]. A recent publication reviewed and compared clinical guidelines issued by various international scientific societies, underscoring their considerable heterogeneity [4]. This observation was echoed in our own experience following a meeting with a multidisciplinary group of adult myositis experts—including internists and rheumatologists—from the *Catalan Clinical Excellence Units Network* (XUEC, Xarxa de Unitats d’Expertesa Clínica) [5]. In this article, we review the current evidence and strategies derived from established management guidelines and analyze their application in clinical practice.

## Classification and Phenotypes in Inflammatory Myopathy

To establish a logical and comprehensive approach to IM, a robust classification system for these heterogeneous diseases is essential. Since the initial classification proposed by Bohan and Peter in 1976 [6], several efforts have been made to refine this framework. One of the most widely used in clinical research is the 2017 EULAR/ACR classification [7]. However, this system has notable limitations, such as the incomplete criteria for immune-mediated necrotizing myopathy (IMNM) and inclusion body myositis (IBM), as well as the omission of numerous autoantibodies identified since its publication [8].

These limitations have led to the development of more specific classification criteria for the main disease entities within the inflammatory myopathy spectrum: dermatomyositis (DM), which presents with various forms or phenotypes [9]; polymyositis (PM), now considered an exclusion diagnosis and an exceptionally rare condition; IMNM [10]; overlap syndromes (OS), which primarily include anti-synthetase syndrome (ASyS) [11, 12] and scleromyositis (SclMyo); and IBM [13], which remains refractory to both immunosuppressive and non-immunosuppressive therapies.

In parallel with these refined disease definitions, the characterization of distinct clinical phenotypes—based on clinical presentation, muscle pathology, and autoantibody profiles—has proven invaluable. This phenotypic approach supports individualized patient assessment and facilitates optimal therapeutic decision-making. Furthermore, it provides a solid foundation for designing clinical trials aimed at generating high-quality evidence to improve outcomes for patients with myositis.

Illustrative examples within DM include anti-MDA5–positive patients with clinically amyopathic DM and rapidly progressive interstitial lung disease; patients with anti-TIF1γ antibodies, who have a strong association with malignancy; and those with classic cutaneous features and anti-Mi2 positivity, often demonstrating favorable responses to immunosuppressive therapy. Similarly, IMNM can be subclassified based on the presence of specific autoantibodies, such as anti-HMGCR—frequently associated with statin exposure—or anti-SRP. Patients negative for both markers are categorized as having seronegative IMNM.

In ASyS, diverse autoantibodies delineate distinct clinical manifestations, while in SclMyo, the presence of anti-PM/Scl or anti-Ku antibodies helps define its clinical and immunological profile within the overlap syndrome spectrum.

The following section of this manuscript presents diagnostic and therapeutic algorithms tailored to the various clinical manifestations, based on the current understanding of IM subtypes, phenotypes, and autoantibody profile.

## Diet and Lifestyle

Few studies have specifically examined diet as an etiopathogenic factor in idiopathic myositis (IM). Rare dietary triggers have been described, including certain herbal supplements such as spirulina (a blue-green algae) [14] and gluten in the context of celiac disease [15, 16]. Some supplements, notably creatine, may support muscle metabolism and have shown benefit when combined with exercise [17]; however, creatine is not routinely recommended in current clinical practice. Diets containing natural statin sources—such as red yeast rice (lovastatin) [18] or certain mushrooms rich in pravastatin [19]—may precipitate disease flares or contribute to IMNM pathogenesis, particularly in individuals with anti-HMGCR autoantibodies. Although the Mediterranean diet, known for its antioxidant and anti-inflammatory properties, is generally recommended for patients with myositis—as it is for the general population—there is currently insufficient evidence to support its specific efficacy in this patient group [20].

Lifestyle measures are also important. Sun avoidance is recommended, especially in DM, as ultraviolet light can exacerbate disease activity [21]. Protective strategies include high-SPF sunscreen (>50) and sun-protective clothing. Vaccination is strongly encouraged, particularly against influenza, SARS-CoV-2, hepatitis B, pneumococcus, and herpes zoster—the latter especially in patients receiving JAK inhibitors [22]. Screening for latent tuberculosis with an interferon-γ release assay is also recommended.

Finally, moderate exercise should be encouraged to prevent immobilization-related atrophy and for its anti-inflammatory benefits. Exercise has demonstrated clear efficacy in patients with myositis and should be initiated early, including during active disease [23].

## Disease Manifestations

### Skeletal Muscle Inflammation and Refractory Myositis

Treatment recommendations from the guidelines until now published [3, 24–27] include the use of glucocorticoids in different doses (1 mg/kg/d or i.v pulses of 500 mg to 1 gr for 3–5 days), with an optional addition of classical (methotrexate or azathioprine) or new immunosuppressive agents (mycophenolate mofetil or calcineurin inhibitors). In refractory cases, where patients do not respond adequately after 3 months of therapy with glucocorticoids and an immunosuppressive agent, alternative treatments such as cyclophosphamide, rituximab, or monthly intravenous immunoglobulin (IVIG) are considered (see supplementary Table 1S for dose, administration, indications and adverse events). Plasma exchange therapy and CAR T cells therapy are treatments that must be used in desperate situations. However, a recent Cochrane Library review on targeted immunosuppressive and immunomodulatory therapies for idiopathic inflammatory myopathies, which included 16 studies with a total of 830 participants, did not find moderate- or high-certainty evidence of benefit compared to placebo. Specifically, there was no significant improvement in functional outcomes, disability, or muscle strength after a minimum follow-up period of six months [28].

In our clinical practice, glucocorticoids combined with immunosuppressive agents are generally the first-line treatment at the onset of disease (see Algorithm 1). The choice of immunosuppressive agent is tailored to the individual patient’s disease characteristics and personal factors. For instance, in young women, we prefer calcineurin inhibitors over mycophenolate mofetil due to the potential risk of embryopathy with accidental pregnancy in females on mycophenolate mofetil therapy. In elderly patients with a high cardiovascular risk profile, we avoid calcineurin inhibitors due to their potential to induce hypertension and hyperglycemia, opting instead for mycophenolate mofetil. While methotrexate is a well-established and effective immunosuppressive agent in myositis, particularly in the presence of severe joint disease, we generally avoid its use in patients with concomitant interstitial lung disease (ILD) due to concerns about potential lung toxicity.

After a thorough clinical evaluation of muscle involvement, if there is no improvement in creatine kinase levels and muscle weakness during the first few weeks (refractory myositis) [29], we initially add IVIG, followed by rituximab, sometimes simultaneously. In cases where the disease is extremely severe from the outset, IVIG is included in the initial treatment strategy.

The choice of treatment may also vary depending on the specific phenotype or type of IM. For patients diagnosed with IMNM who are positive for anti-HMGCR or anti-SRP antibodies, we typically combine glucocorticoid therapy with additional agents such as methotrexate, rituximab, and/or IVIG. Plasma exchange therapy is also considered in extreme cases [30–33]. Although experience is limited, CAR T-cell therapy appears to be a promising option for refractory cases of IMNM or ASyS [34].

### Skin Rash, Cutaneous Ulcers, Panniculitis, and Calcinosis

Cutaneous involvement in IM is heterogeneous and, in some cases, may constitute the sole clinical manifestation, even in the absence of muscle weakness, as is observed in amyopathic DM. For evaluating the severity and extent of skin involvement in patients with DM, we use the *Cutaneous Dermatomyositis Disease Area and Severity Index (CDASI)* [35]. This internationally validated instrument is widely applied in both clinical practice and clinical trials to provide a standardized and reliable assessment of cutaneous disease activity. Immunological profile provides additional diagnostic and prognostic value by helping to predict the pattern and severity of skin lesions. For example, anti-Mi-2–associated lesions—such as Gottron’s papules and macules or heliotrope rash—typically respond favorably to glucocorticoid therapy, whereas anti-MDA5–associated ulcers in clinically amyopathic DM often present significant therapeutic challenges.

Current guidelines [24–27] recommend sun protection, topical glucocorticoids, or topical calcineurin inhibitors (e.g., tacrolimus or pimecrolimus) frequently in combination with oral antimalarials. In our clinical practice, we work closely with dermatologists, and we generally do not favor the use of antimalarials due to limited efficacy and the frequent occurrence of toxic or allergic reactions [36]. While antimalarials are often beneficial for cutaneous and mild articular manifestations in lupus, our experience with DM has been less favorable.

In cases characterized by extensive skin disease, chronicity, or refractory ulcers, escalation to systemic immunosuppressive therapy is warranted. Agents reported in medical bibliography include azathioprine, methotrexate, calcineurin inhibitors, and mycophenolate mofetil, while IVIG and rituximab are typically reserved for severe or treatment-refractory cases. Recent therapeutic advances include Janus kinase inhibitors (JAKi) and biologic agents such as anifrolumab—a monoclonal antibody targeting the type I interferon receptor—and dazukibart—a neutralizing monoclonal antibody against IFNβ—both aiming to correct the type I interferon dysregulation characteristic of myositis [37, 38].

Ulcers associated with anti-MDA5 antibodies remain particularly resistant to standard therapy. In selected cases, the addition of endothelin receptor antagonists (e.g., bosentan) or phosphodiesterase inhibitors (e.g., sildenafil, apremilast) has been associated with ulcer resolution [39–41]. Information is summarized in Algorithm 2.

Panniculitis, an inflammatory process affecting the adipose panniculus located between the skin and muscle, may also occur in IMs, particularly in DM [42, 43]. Clinically, it presents as painful subcutaneous red nodules, most frequently on the arms and legs. Histopathological examination usually demonstrates lobular panniculitis occasionally with lymphocytic vasculitis and abundant mucin interstitially deposited in the dermis. Associations have been described with specific autoantibodies, notably anti-MDA5 [44] and anti-SAE [45], in DM patients. Clinical manifestations often improve following the initiation of glucocorticoids and immunosuppressive agents, especially tacrolimus, or more recently JAKi. Nonetheless, the inflammatory process may progress to atrophy—most often lipoatrophy, more frequent in juvenile forms but also observed in adults—or to calcification, representing pathological sequelae of the healing process, commonly referred to as calcinosis.

Calcinosis, particularly severe forms such as *calcinosis universalis* or *milk calcinosis*, can be extremely difficult to manage [46, 47]. Existing guidelines rarely address this manifestation in detail. In our clinical practice, therapeutic approaches include intensification of immunosuppression with agents such as rituximab or JAKi, the use of bisphosphonates, calcium channel blockers, or topical sodium thiosulfate administration (Table 1). However, clinical responses are often suboptimal, and surgical intervention occasionally may be required, particularly in the presence of compartment syndrome or when large calcific masses compromise vital structures. A summary of our therapeutic approach is provided in Algorithm 3.

### Interstitial Lung Disease

Interstitial lung disease (ILD) is a common complication in patients with IM and may present with diverse clinical patterns. Autoantibody profile is essential for identifying the main clinical phenotypes [48]. Certain myositis-specific autoantibodies define well-recognized syndromes, such as the ASyS, for which robust classification criteria are now available [11]. Other autoantibodies, including anti-Ku or anti-PM/Scl in SclMyo and anti-RNP in mixed connective tissue disease (Sharp syndrome), are characteristic of overlap syndromes. All these forms generally have an insidious onset, allowing time for clinicians to establish an accurate diagnosis and initiate immunosuppressive therapy. The presence of the myositis-associated autoantibody anti-Ro52 has been consistently linked to a poorer prognosis across all phenotypes [49].

In contrast, certain ILD presentations are markedly more severe, notably the rapidly progressive ILD associated with clinically amyopathic DM and anti-MDA5 antibodies. In such cases, the therapeutic strategy must be intensified and may include, in addition to conventional immunosuppressive agents—particularly mycophenolate mofetil and calcineurin inhibitors—B-cell–depleting therapy with rituximab or Janus kinase inhibitors such as tofacitinib. In specialized centers, additional interventions such as plasma exchange combined with IVIG or, in selected cases, extracorporeal membrane oxygenation (ECMO) may be considered, either as a bridge to lung transplantation or while awaiting the therapeutic effects of immunosuppressive treatment [33, 50].

**Fig. 1:**
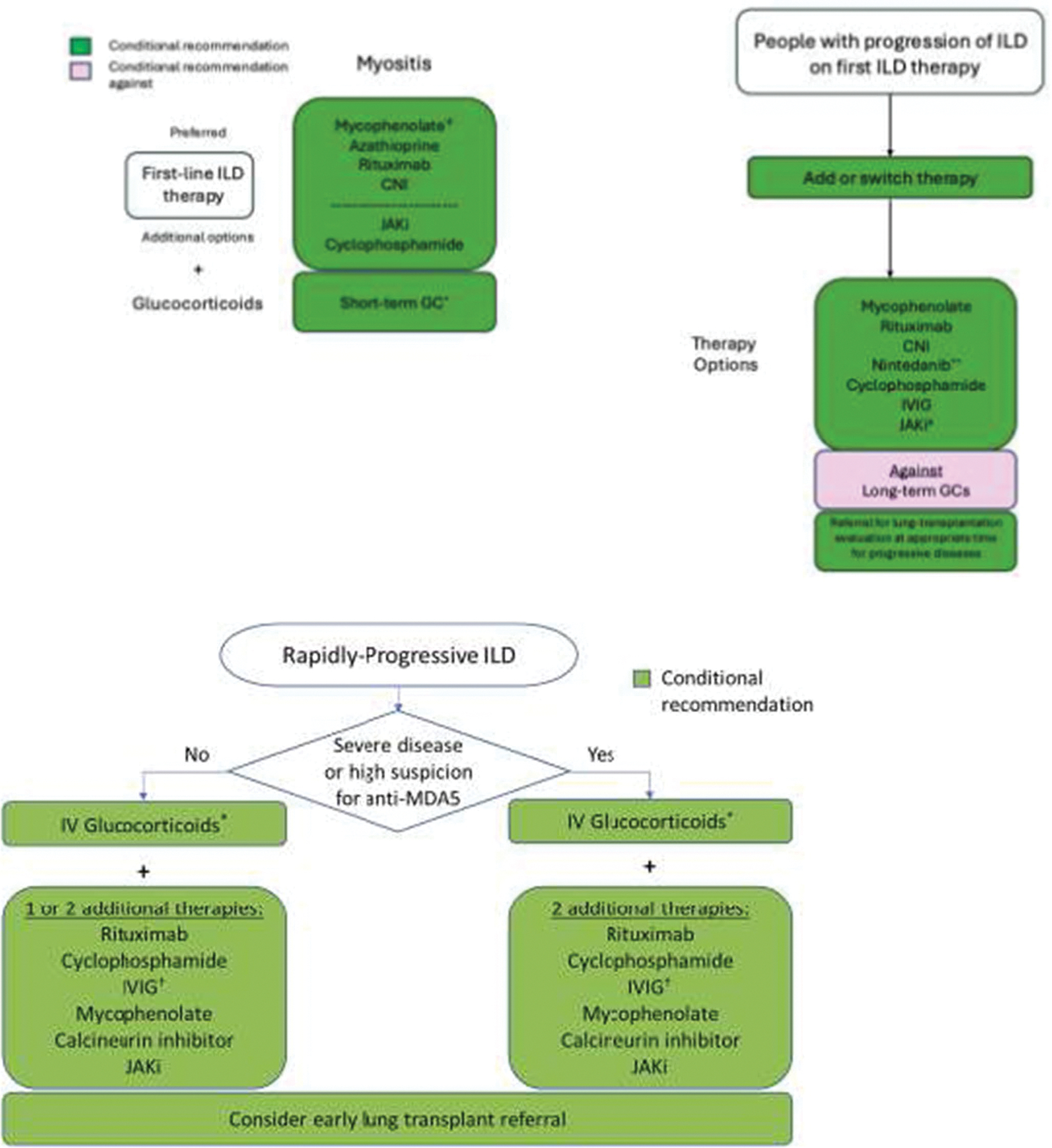
First-line treatment and management of ILD progression and RP-ILD in patients with IIM-ILD. Modified from 2023 American College of Rheumatology (ACR)/American College of Chest Physicians (CHEST) Guideline for the Treatment of Interstitial Lung Disease in People with Systemic Autoimmune Rheumatic Diseases [52]. *Short-term is defined as 3 months or less. ^†^ Treatments are listed in order based on a hierarchy established by head-to-head votes, although the panel noted that decisions on which first-line therapy to use were dependent on specific situations and patient factors. Mycophenolate was conditionally recommended over the other listed therapies. Therapies are divided into “preferred” and “additional” options based on the rank-order hierarchy. ^#^JAKi conditionally recommended as an option particularly in patients with anti–MDA-5. ** Decision on use of nintedanib vs. immunosuppression depends on pace of progression and amount of fibrotic disease or presence of a usual interstitial pneumonia pattern on CT chest. CNI, calcineurin inhibitor; CT, computed tomography; GC, glucocorticoid; ILD, interstitial lung disease; IVIG, intravenous immunoglobulin. JAKi, JAK inhibito

A recent comprehensive review has addressed this topic in detail, and specific guidelines for the management of ILD in myositis have been published [51, 52]. According to these guidelines, first-line therapy consists of short-term glucocorticoids, with the dosage and duration tailored to disease severity. This should be followed by the introduction of one of the recommended immunosuppressive agents, selected according to the hierarchical order established by expert consensus: mycophenolate mofetil, azathioprine, rituximab, calcineurin inhibitors, and, as additional options, JAKi or cyclophosphamide. Additional general and disease-specific management recommendations outlined in the guidelines are summarized in Table 2; Fig. 1.

In our clinical practice [33, 50, 53, 54], we distinguish between rapidly progressive forms—most frequently associated with anti-MDA5 antibodies—and more chronic disease patterns, which are typically linked to the ASyS or overlap syndromes. This distinction enables more targeted and timely therapeutic decision-making. In cases of rapidly progressive ILD, the detection of anti-MDA5 antibodies is particularly valuable for defining the clinical scenario and confirming the diagnosis, especially when both clinical and radiological deterioration occur steadily within the first month, a hallmark of the rapidly progressive form. Our initial treatment strategy usually combines glucocorticoids (1 mg/kg/day orally, or intravenous pulses of 500 mg–1 g/day for the first three days) with calcineurin inhibitors, most often tacrolimus (2–3 mg every 12 h, maintaining blood levels between 4 and 12 mcg/mL). If tacrolimus is contraindicated or poorly tolerated, we switch to mycophenolate mofetil (1 g every 12 h). Clinical monitoring is performed in close collaboration with pulmonologists and ICU specialists. If arterial blood gas analysis or overall clinical status worsens, we promptly add tofacitinib; in some cases, tacrolimus and tofacitinib are initiated simultaneously, generally with good tolerance. In the most severe presentations, early use of polymyxin B hemoperfusion and plasma exchange with IVIG replacement appears to be beneficial. Prophylaxis with cotrimoxazole is instituted from the outset to reduce the risk of *Pneumocystis jirovecii* infection. When indicated by ICU specialists, ECMO is considered a valuable bridge—to allow time for immunosuppressive therapy to take effect, to facilitate a change in therapeutic strategy, to support the patient until response to rituximab can be assessed, or to maintain clinical stability during lung transplantation evaluation and, if feasible, transplantation itself. Algorithm 4 summarizes this therapeutic approach.

In patients with overlap syndrome–associated ILD or the more common ASyS, our initial approach typically involves tacrolimus or mycophenolate, with close monitoring of clinical status and pulmonary function tests. Disease progression is defined as a decline in forced vital capacity (FVC), and change/progression in extend ILD by high-resolution computed tomography.

In such cases, we generally add rituximab to the existing immunosuppressive regimen and reassess the patient after six months. If progression persists despite this combination therapy, the addition of a JAKi, most often tofacitinib (5 mg every 12 h), is our next therapeutic step. Decision-making is guided by a multidisciplinary ILD committee, which includes pathologists, radiologists, pulmonologists, internists, and rheumatologists. These discussions aim to determine whether deterioration is driven by ongoing inflammatory activity—requiring escalation of immunosuppressive therapy—or by progressive fibrotic ILD, which warrants the addition of an antifibrotic agent such as nintedanib or pirfenidone. Algorithm 5 summarizes this treatment pathway.

### Ventilatory failure

Ventilatory failure in patients with IM is primarily related to diaphragmatic involvement. Although the diaphragm, as a skeletal muscle, may be affected by the underlying disease, its impairment usually parallels overall musculoskeletal activity and generally responds to conventional immunosuppressive therapy, rarely representing a major clinical problem. Nevertheless, approximately 5% of patients develop severe diaphragmatic dysfunction, which can lead to ventilatory failure. Certain clinical and functional indicators may help to identify these patients, including elevated arterial carbon dioxide tension (hypercapnia), a FVC < 50% in the absence of ILD, or a >20% decline in FVC from the upright to the supine position. In such cases, treatment with high-dose glucocorticoids in combination with IVIG and a calcineurin inhibitor may be required to control respiratory failure. While awaiting therapeutic response, non-invasive ventilation may be necessary and can be lifesaving [55].

### Dysphagia

Dysphagia is a common manifestation in patients with myositis and may develop through two distinct pathophysiological mechanisms. First, involvement of smooth muscle within the gastrointestinal tract, particularly the esophagus, can lead to a pattern of dysphagia similar to that observed in systemic sclerosis. This mechanism is characteristically present in certain myositis phenotypes, especially the ASyS and SclMyo [56], but not in others, such as IMNM. A second mechanism involves dysfunction of the cricopharyngeal muscle, a striated muscle that plays a pivotal role in initiating the swallowing process [57]. Although cricopharyngeal involvement is relatively frequent in myositis, it is generally mild and tends to respond to conventional immunosuppressive therapy. Nonetheless, a small subset of patients may develop severe dysphagia requiring not only aggressive immunosuppressive treatment—particularly IVIG—but also temporary enteral nutritional support via nasogastric tube. Severe dysphagia may lead to serious complications, including aspiration pneumonia, interstitial lung disease, and malnutrition. Given the absence of established guidelines for the management of this complication, we propose a rational therapeutic algorithm to address severe dysphagia in myositis, as outlined in Algorithm 6.

Several key points should be emphasized. Videofluoroscopy remains the gold standard for diagnosing dysphagia secondary to cricopharyngeal involvement. Intravenous immunoglobulin should be incorporated into the immunosuppressive regimen whenever feasible, given its demonstrated efficacy in improving this manifestation. Close collaboration with speech-language pathologists and nutritionists is highly beneficial, as the implementation of structured rehabilitation protocols is of paramount importance for clinical improvement. In patients unresponsive to immunosuppressive therapy, enteral nutritional support—either via nasogastric tube or percutaneous endoscopic gastrostomy—is mandatory. It is crucial to reassure patients that, in many cases, clinical improvement occurs within 3–6 months, allowing withdrawal of enteral feeding. Only in highly selected cases, such as those with a cricopharyngeal bar, more invasive interventions (e.g., botulinum toxin injection, cricopharyngeal myotomy, or esophageal balloon dilatation) may be considered, although reported outcomes remain inconsistent.

### Myocarditis

Myocarditis, once considered a rare manifestation of IM, is now recognized as having greater clinical significance than previously anticipated. Although it may remain asymptomatic and clinically silent for years, cardiac involvement is associated with an increased risk of mortality due to arrhythmias, systolic dysfunction, or heart failure. Several biomarkers and imaging modalities have been proposed for the evaluation of cardiac inflammation, including high-sensitivity troponin I, NT-proBNP, electrocardiography, transthoracic echocardiography (TTE), cardiac magnetic resonance imaging (CMR), and nuclear imaging techniques such as ^18FDG-PET/CT and myocardial perfusion SPECT. Nevertheless, endomyocardial biopsy (EMB) remains the diagnostic gold standard and is required in selected cases [58, 59].

**Fig. 2:**
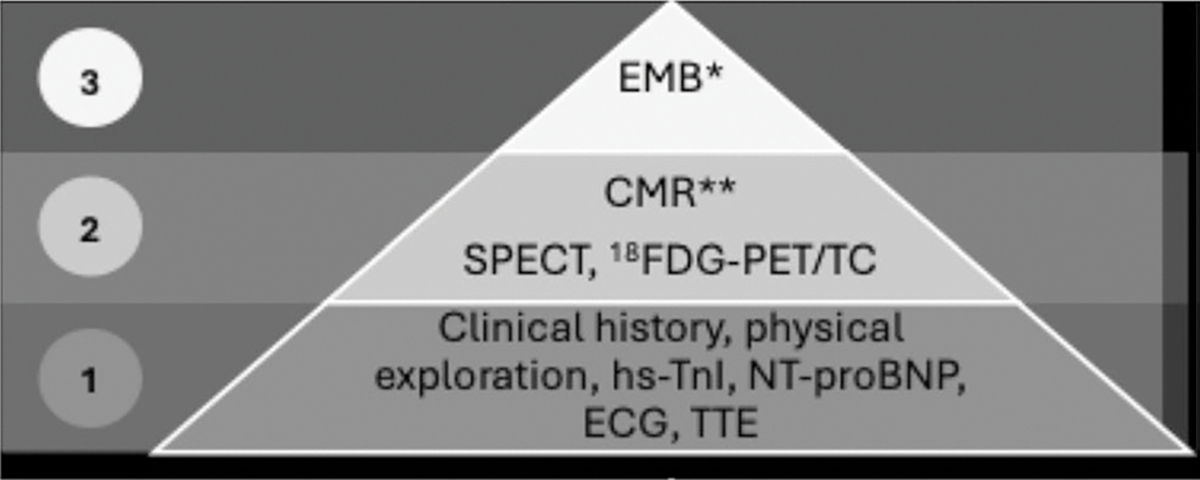
Diagnostic algorithm for myocarditis in IIM. *Gold-standard invasive technique. **Gold-standard non-invasive technique. EMB, endomyocardial biopsy; CMR, cardiac magnetic resonance; SPECT, single-photon emission computed tomography; 18FDG-PET/CT, positron emission tomography with 18Fluorodeoxyglucose and computed tomography; hs-TnI, high-sensitivity troponin I; NT-proBNP, N-terminal prohormone of brain natriuretic peptide; ECG, electrocardiogram; TTE, transthoracic echocardiography

Currently, no international guidelines exist for the systematic screening of these patients. Evidence from small, single-center studies has suggested potential risk factors for myocardial involvement in IM, including seropositivity for anti-SRP, antimitochondrial antibodies, anti-MDA5, or anti-synthetase antibodies [60–65]. A pragmatic diagnostic strategy involves an initial assessment with a detailed clinical history, physical examination, and measurement of myocardial injury biomarkers (high-sensitivity troponin I and NT-proBNP), along with electrocardiography and TTE. CMR should subsequently be performed in patients at high risk or in those with abnormal findings on initial testing (Fig. 2), as it is currently considered the noninvasive reference standard. EMB should be reserved for cases in which the etiological diagnosis remains uncertain, particularly when viral myocarditis is suspected.

**Fig. 3:**
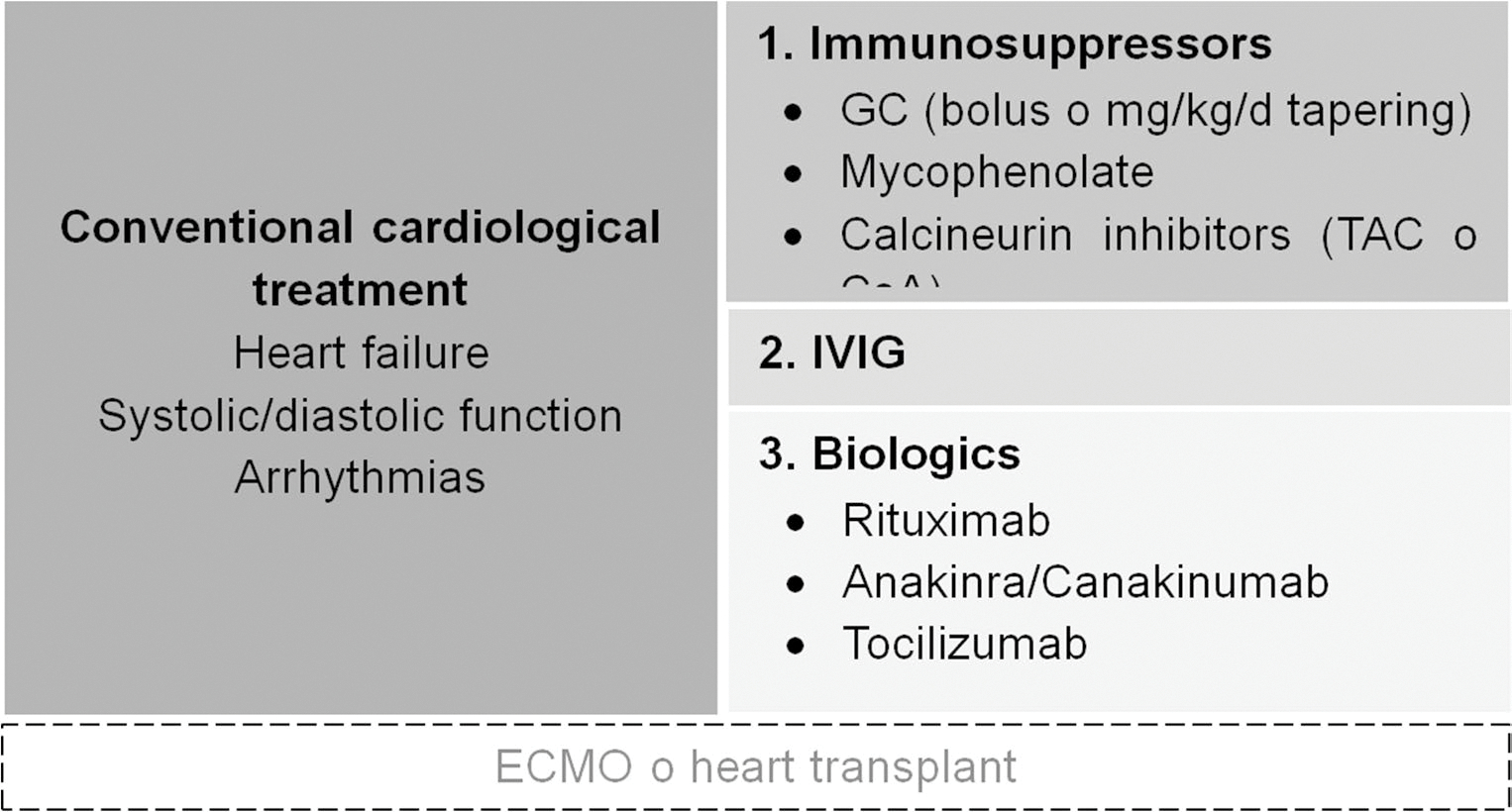
Treatment algorithm for myocarditis in inflammatory myopathy. ECMO: extracorporeal membrane oxygenation, GC, glucocorticoids; TAC, tacrolimus; CsA, cyclosporine

Beyond cardiology-specific management of heart failure and arrhythmias, no formal treatment guidelines currently exist for myocarditis in IM. Therapeutic strategies are generally extrapolated from the management of myocarditis in other systemic diseases and from general therapeutical principles applied in myositis, with immunosuppression remaining the cornerstone of therapy to reduce myocardial inflammation. However, the use of glucocorticoids, immunosuppressants such as mycophenolate mofetil or tacrolimus, intravenous immunoglobulins, and biologic agents such as rituximab has shown inconsistent efficacy in halting the inflammatory process. Although no dedicated studies have specifically addressed IM-associated myocarditis, recent evidence in refractory myocarditis highlights a pivotal role for interleukin-1 (IL-1) in disease pathogenesis. Consequently, IL-1 receptor antagonists (e.g., anakinra or canakinumab) have emerged as promising therapeutic options [66]. In highly refractory cases, extracorporeal membrane oxygenation (ECMO) or heart transplantation may be considered as rescue strategies (Fig. 3).

### Arthritis

Arthritis is a common clinical manifestation in IM, particularly in certain phenotypes such as ASyS and DM associated with anti-MDA5 antibodies. In ASyS, arthritis may occasionally predominate in the clinical picture and, in some cases, precede other major manifestations such as ILD or myositis. Under these circumstances, patients may be misdiagnosed with seronegative rheumatoid arthritis (RA). Typically, arthritis in IM presents as a symmetric, non-erosive polyarthritis, predominantly affecting large joints as well as the small joints of the hands (metacarpophalangeal and proximal interphalangeal) and feet. Less commonly, it may present as oligoarthritis or asymmetric polyarthritis. The assessment of rheumatoid factor and anti-cyclic citrullinated peptide antibodies is essential in patients with IM and arthritis, as the coexistence of IM and RA has been well documented and represents a recognized overlap syndrome [67].

In the absence of specific studies addressing the treatment of arthritis in IM, management generally follows therapeutic strategies established for RA. Notably, with the exception of glucocorticoids, most conventional immunosuppressants commonly used to treat major IM manifestations such as myositis or ILD—including azathioprine, mycophenolate mofetil, and calcineurin inhibitors—have limited efficacy in controlling arthritis. When arthritis is the predominant manifestation and ILD is absent, methotrexate (MTX) is usually considered first-line therapy, combined with glucocorticoids (at the lowest effective dose and for the shortest possible duration) and nonsteroidal anti-inflammatory drugs, administered transiently as bridging therapy until MTX becomes effective. The main concern regarding MTX in patients with concomitant ILD is the risk of acute pneumonitis. This rare complication, typically occurring within the first year of treatment, is believed to result from a hypersensitivity reaction rather than cumulative dose toxicity [68].

For this reason, in patients with ILD, leflunomide is often considered a safer alternative. Regarding its safety, the potential association between leflunomide and ILD appears to be restricted to patients of Asian ancestry, according to two post-marketing pharmacovigilance studies conducted in Japan and Korea [69, 70].

In cases of inadequate response to conventional therapy, rituximab (RTX) is the biologic agent with the strongest supporting evidence, particularly in patients with concomitant ILD. In the absence of ILD, or in patients with mild or stable ILD, other biologic therapies with proven efficacy in RA—such as JAK inhibitors, abatacept, or IL-6 inhibitors—may be considered. By contrast, TNF inhibitors are generally discouraged, as cases of TNF inhibitor–induced myositis have been reported in RA patients [71]. All these data are summarized in Algorithm 7. A more general information is depicted in Algorithm 8 [2].

## Randomized, Placebo-Controlled Clinical Trials in Myositis

Evidence-based therapies for patients with IM remain limited, as highlighted in a recent Cochrane review on targeted therapies addressing immunological pathways [28]. Below is a summary of the most relevant studies.

### Intravenous immunoglobulins (IVIG)

Although, as mentioned above, evidence on targeted therapies in IIM is scarce, non-targeted therapies—particularly IVIG—have demonstrated some efficacy [72]. Two randomized controlled trials have evaluated the effectiveness of IVIG in dermatomyositis (DM). The first study [73], published in 1993, was a double-blind, placebo-controlled trial that included 15 patients with refractory DM (defined as lack of response to glucocorticoids plus one immunosuppressive agent administered for at least 6 months). Patients were randomized to receive monthly IVIG or placebo for three months, with the option to cross over to the alternative treatment for an additional three months. Those initially assigned to IVIG showed significant improvement in muscle strength (*p* < 0.018), as did patients who crossed over from placebo to IVIG. These improvements were also corroborated by repeated muscle biopsy findings. The second trial [74] was a randomized, placebo-controlled study involving 95 patients with active DM, who were assigned to receive IVIG or placebo every four weeks. Improvement, assessed using the Total Improvement Score, was significantly greater in the IVIG group. The only serious adverse events reported were thromboembolic events, occurring in six patients.

### Rituximab (the RIM study)

The RIM trial enrolled 200 patients with refractory myositis in a prospective, double-blind, randomized design. Participants received rituximab (monoclonal anti-CD20) either in the first two weeks of the trial (early rituximab group) or during the last two weeks (late rituximab group), with both compared against placebo [75]. Although the study did not meet its primary endpoint of demonstrating statistically significant differences between groups, clinical improvement was observed in 83% of patients overall.

### Belimumab

In this multicenter, randomized, double-blind, placebo-controlled trial, 15 patients with refractory idiopathic myositis (dermatomyositis or polymyositis) were assigned to receive either placebo or belimumab (10 mg/kg), a fully human IgG1 monoclonal antibody targeting BAFF, in addition to standard of care. No significant differences were observed between the two groups [76].

### Abatacept

Abatacept, a recombinant fusion protein composed of the extracellular domain of human CTLA-4 linked to a fragment of the Fc region of human IgG1, did not demonstrate significant efficacy in a randomized, double-blind, placebo-controlled phase III trial. The study enrolled 148 patients with IM who were randomized to receive either abatacept or placebo in addition to standard-of-care treatment. No significant differences were observed in the proportion of responders between the two groups [77].

### Tocilizumab

In a multicenter, randomized, double-blind, placebo-controlled trial, 36 patients with DM or PM were assigned to receive either tocilizumab (8 mg/kg) or placebo. No significant differences in the Total Improvement Score were observed between the two groups [78].

### Ustekinumab

A multicenter, randomized, double-blind, placebo-controlled trial enrolled 51 adult Japanese patients with DM or PM. Participants were assigned to receive either Ustekinumab, a fully human IgG1κ monoclonal antibody targeting IL-12/IL-23, or placebo. No significant differences in efficacy, as assessed by the Total Improvement Score, were observed between the two groups [79].

### Dazukibart

Type I interferon, particularly IFN-β, is known to be elevated in both the blood and skin of patients with DM, with corresponding upregulation of type I IFN-related genes. In a multicenter, randomized, double-blind, placebo-controlled phase 2 trial, 75 patients were assigned to receive either dazukibart, an IFN-β–specific monoclonal antibody, or placebo. Significant clinical improvement was observed, predominantly among patients with skin-predominant disease [38, 80].

## Conclusions

There is an urgent need to standardize the therapeutic approaches for myositis and its related manifestations, enabling physicians to treat patients within the myositis spectrum using the highest level of available evidence. Well-designed randomized controlled trials targeting specific clinical features—such as dysphagia or ILD—or distinct phenotypes, such as anti-MDA5-positive rapidly progressive ILD, may offer more meaningful insights than studies focused solely on broad disease categories like dermatomyositis or polymyositis. In this review, we aimed to summarize the main therapeutic strategies based on both our clinical experience and the evidence outlined in current guidelines and literature. We hope this work will be a valuable resource for clinicians and others involved in the care of patients with myositis.

## Supplementary Material

Supplementary Material

**Supplementary Information** The online version contains supplementary material available at https://doi.org/10.1007/s40674-025-00239-5.

## Figures and Tables

**Algorithm 1:**
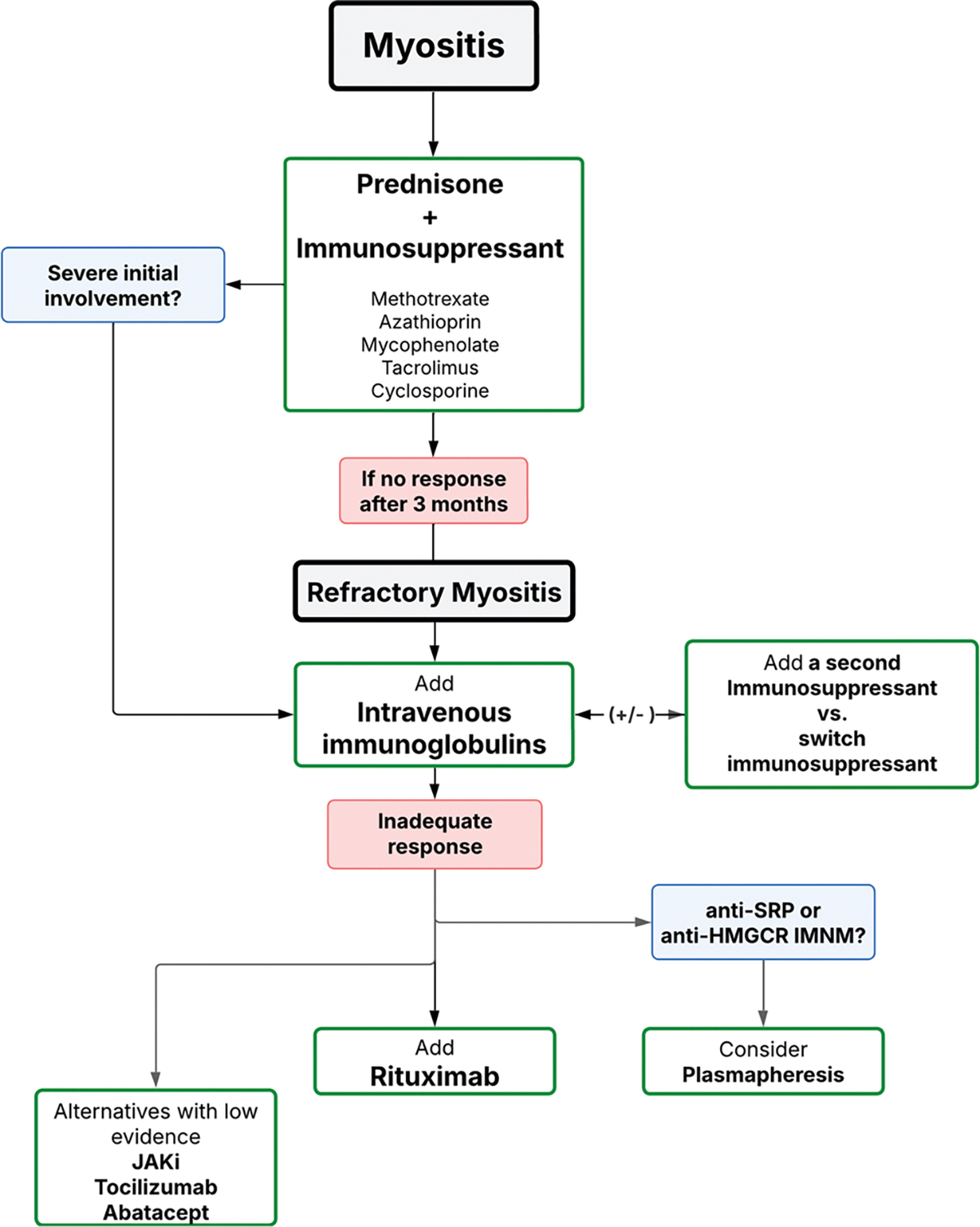
Management of muscle involvement in myositis. IMNM: Immune-mediated necrotizing myopathy. JAKi: JAK inhibitor

**Algorithm 2:**
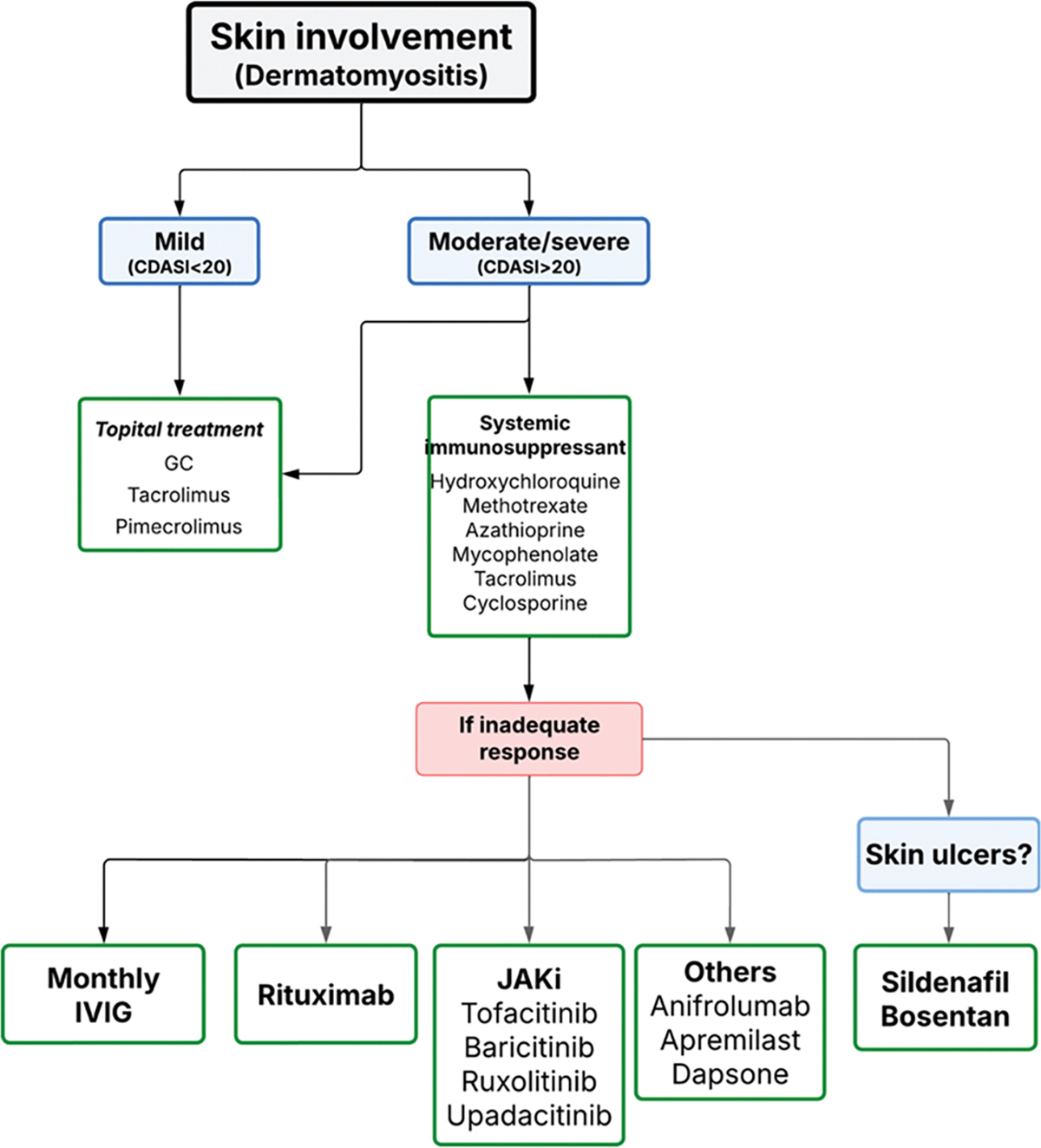
Management of skin involvement in IIM- CDASI: Cutaneous Dermatomyositis Disease Area and Severity Index; GC: Glucocorticoids; IVIG: Intravenous immunoglobulins; JAKi: Janus kinase inhibitors

**Algorithm 3:**
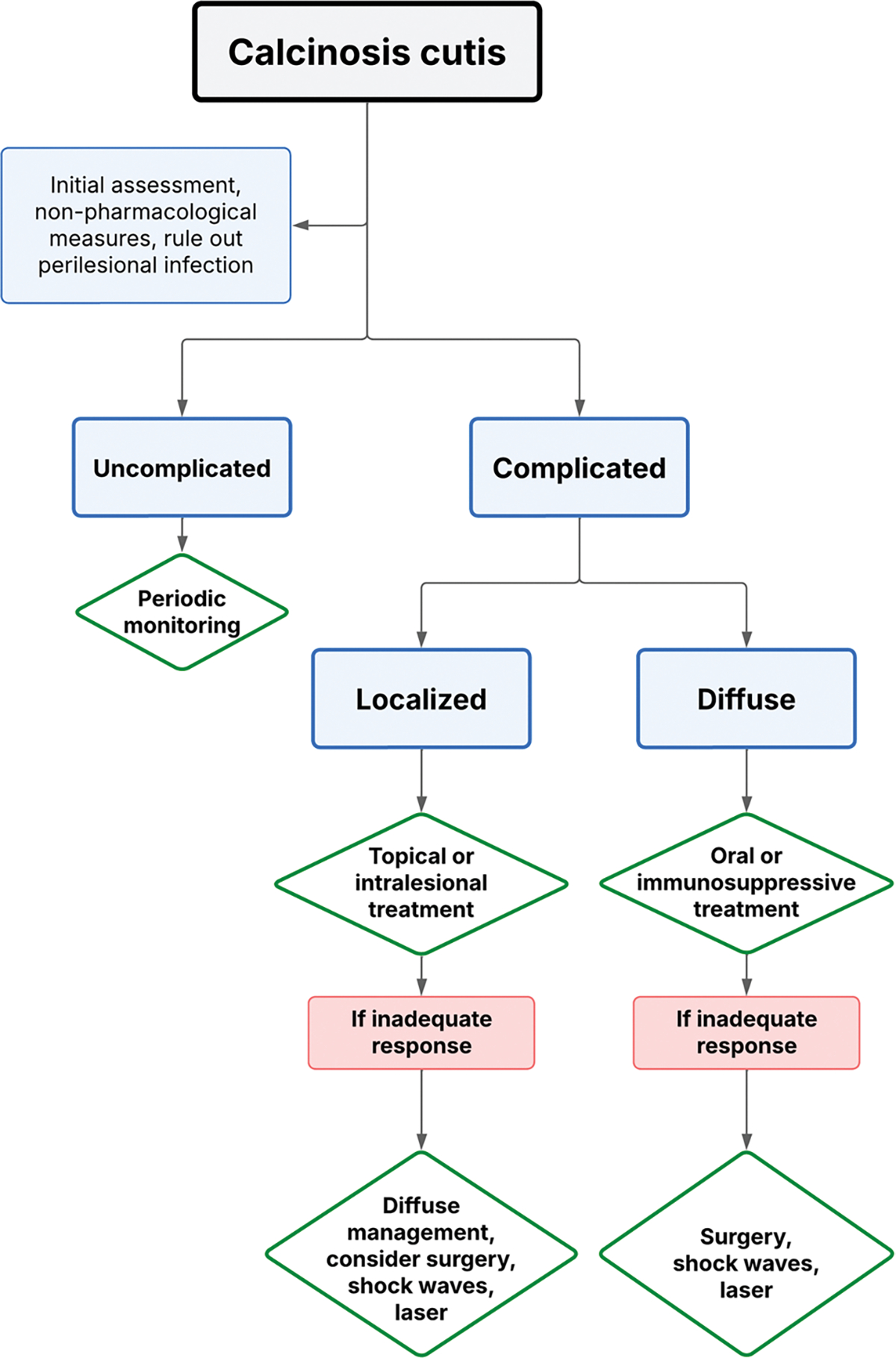
Management of calcinosis cutis

**Algorithm 4:**
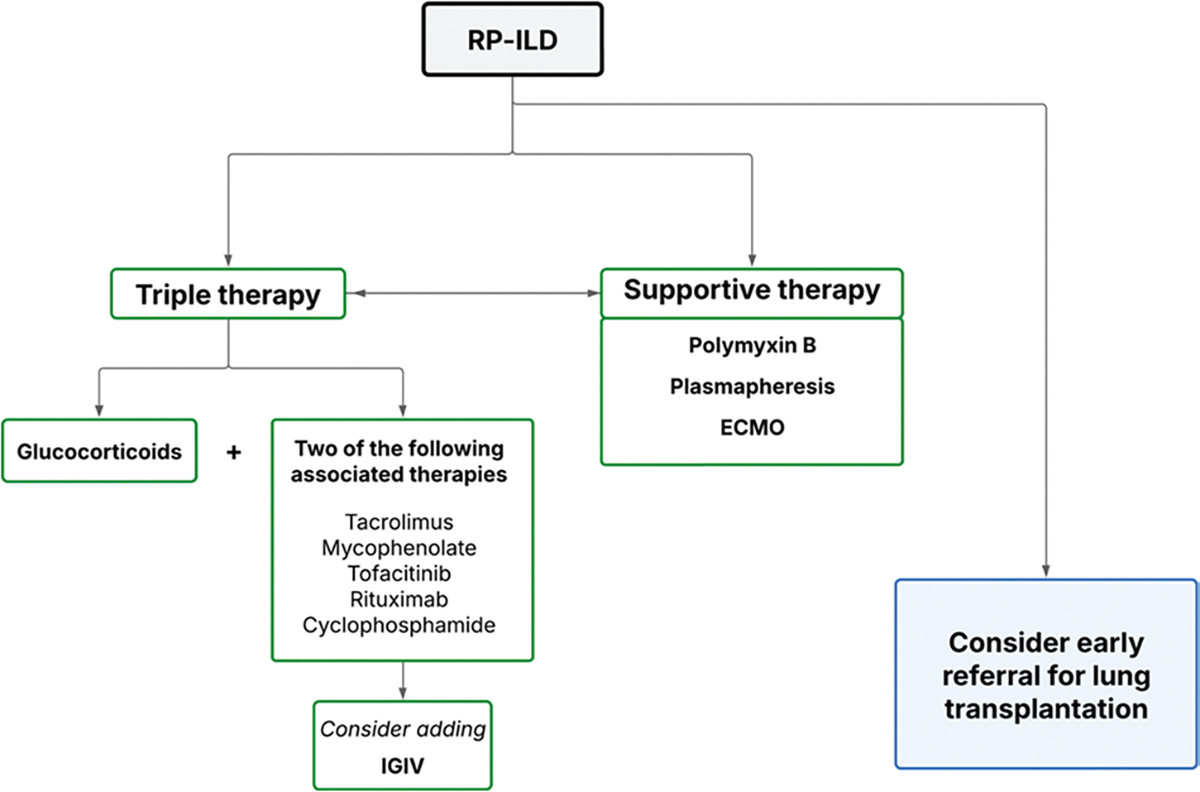
Therapeutic algorithm in rapidly progressive interstitial lung disease RP-ILD: Rapidly progressive interstitial lung disease; IVIG: Intravenous immunoglobulins; ECMO: Extracorporeal membrane oxygenation

**Algorithm 5:**
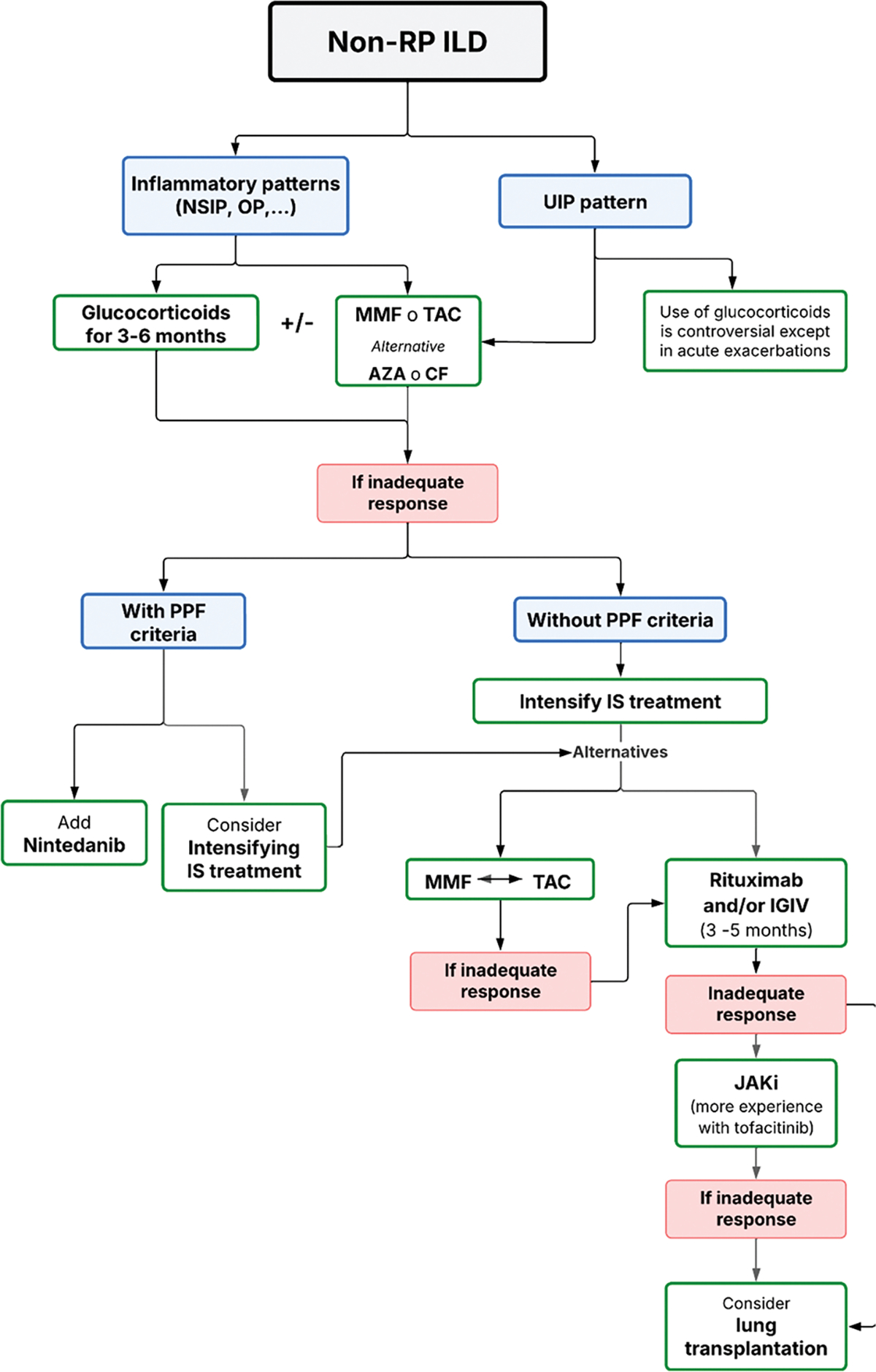
Therapeutic algorithm in non-rapidly progressive interstitial lung disease Non-RP ILD: None rapidly progressive interstitial lung disease; NSIP: Nonspecific interstitial pneumonia; OP: Organizing pneumonia; UIP: Usual interstitial pneumonia; MMF: Mycophenolate mofetil; TAC: Tacrolimus; AZA: Azathioprine; CYC: Cyclophosphamide; PPF: Progressive pulmonary fibrosis; IS: Immunosuppressant; IVIG: Intravenous immunoglobulins; JAKi: Janus kinase inhibitor

**Algorithm 6:**
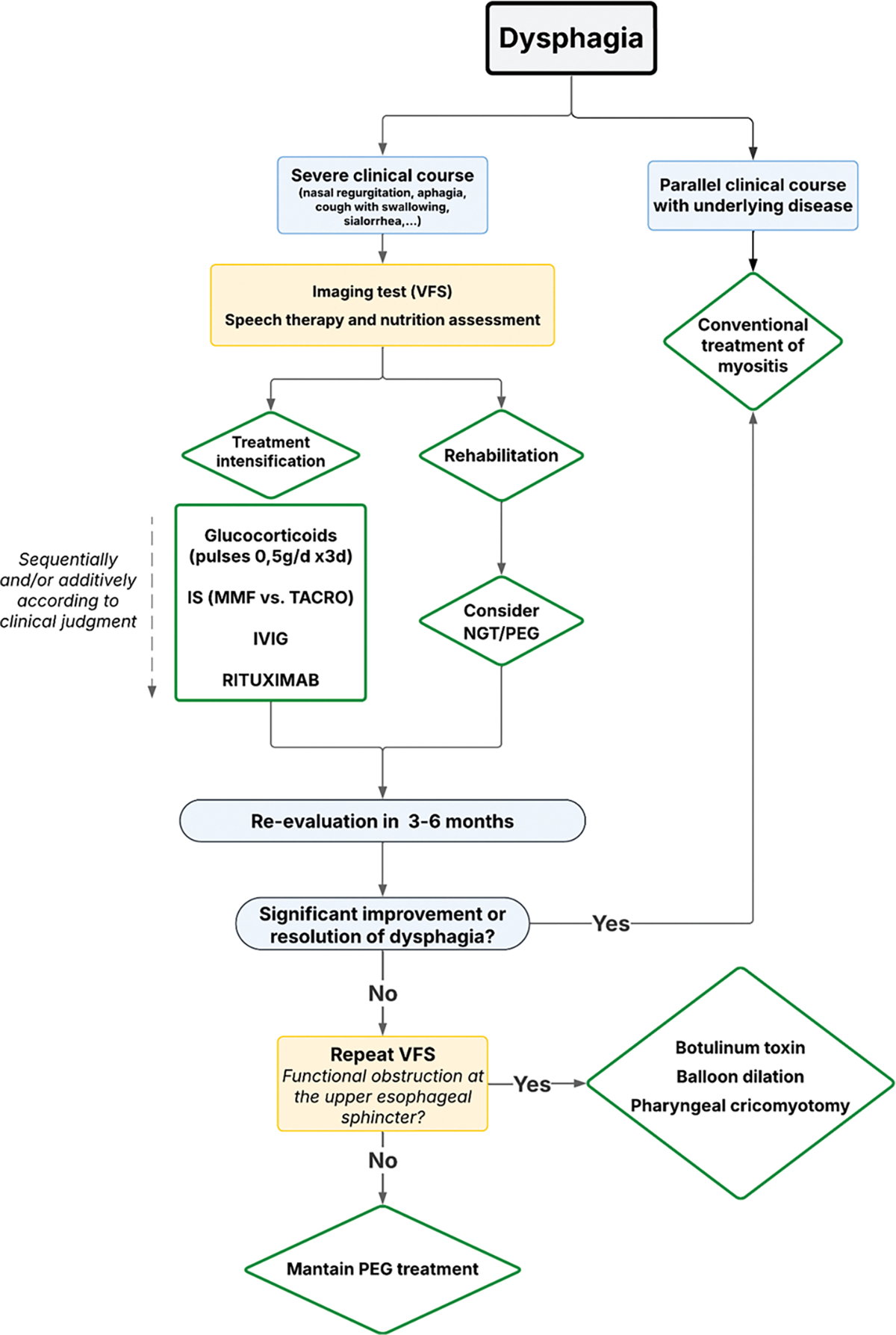
Management of dysphagia. IVIG: Intravenous immunoglobulins; MMF: Mycophenolate mofetil. NGT: Nasogastric tube. PEG: Percutaneous endoscopic gastrostomy. TACRO: Tacrolimus. VFS: Videofluoroscopic swallow study

**Algorithm 7:**
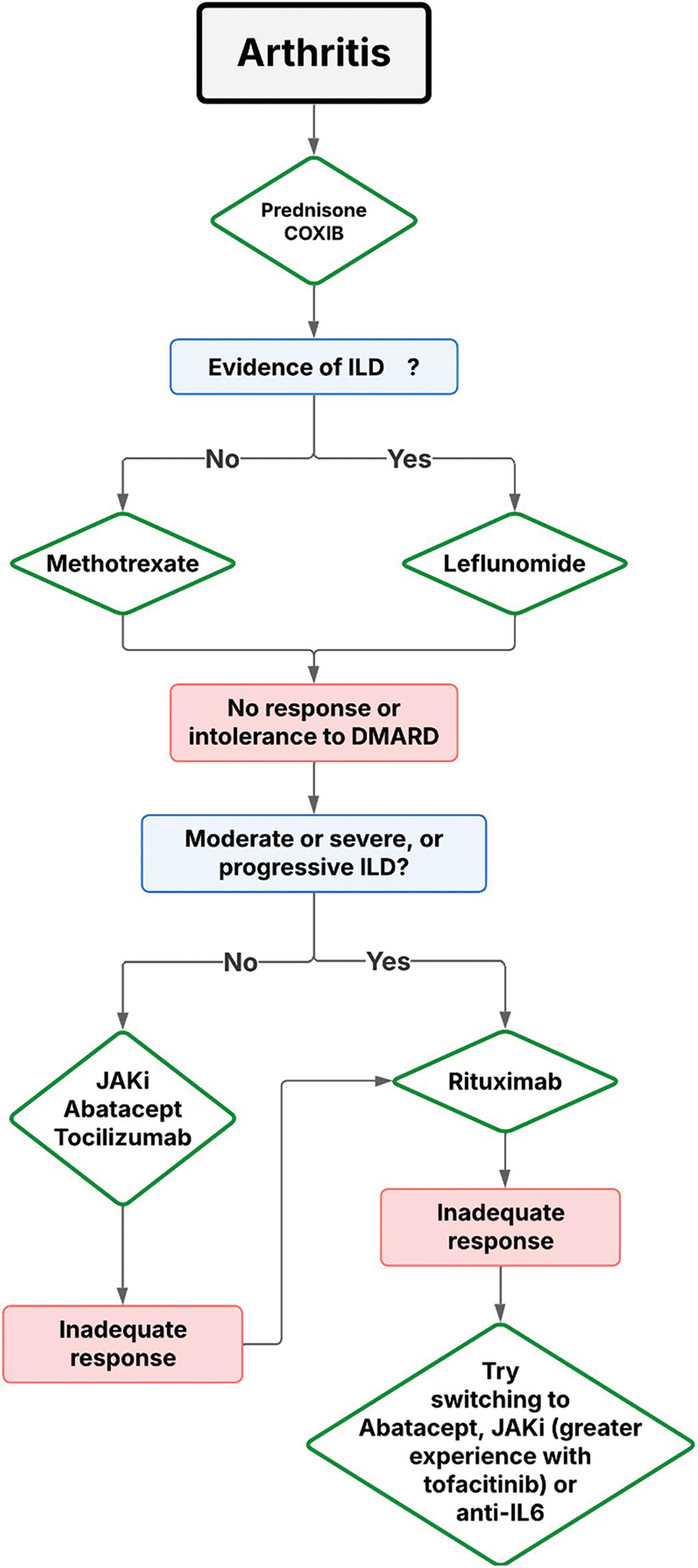
ILD: Interstitial lung disease; DMARD: Disease Modifying Antirheumatic Drugs; JAKi: JAK inhibitors

**Algorithm 8:**
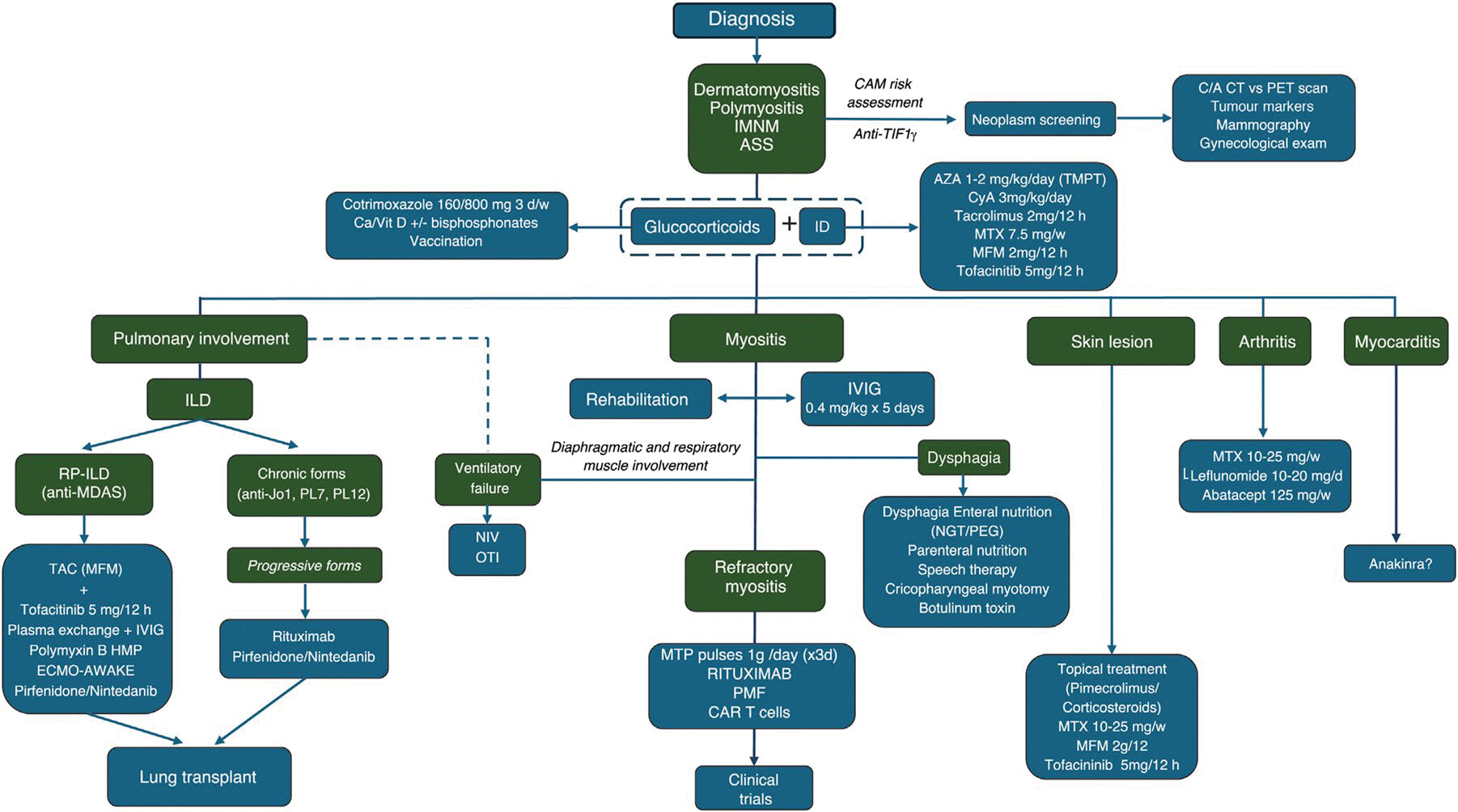
Therapeutic algorithm in patients with idiopathic inflammatory myopathy. ASS: anti-synthetase syndrome; AZA: azathioprine; C/A CT: chest-abdomen CT; CAM: cancer-associated myositis; CyA: cyclosporine; ECMO: extracorporeal membrane oxygenation; GC: glucocorticoids; HMP: hemoperfusion; ILD: diffuse interstitial lung disease; IMNM: immune-mediated necrotizing myopathy; IS: immunosuppressants; IVIG: intravenous immunoglobulins; MFM: mycophenolate mofetil; MTX: methotrexate; NIV: non-invasive ventilation; NGT: nasogastric tube; OTI: orotracheal intubation; PE: plasma exchange; PET: positron emission tomography; RP-ILD: rapidly progressive interstitial lung disease; Tac: tacrolimus; TM: tumor markers; TPMT: thiopurine methyltransferase; W: weekly (adapted from ref. 2)

**Table 1:** Main Pharmacological options in calcinosis cutis treatment

Drug	Mechanism of action	Dose

Sodium thiosulfate	Calcium chelating agent	Topical (25%) b.i.d.
Bisphosphonates	Calcium/phosphor metabolism	Alendronate 70 mg p.o./w
JAKi	Type I interferon pathway and inflammation control	Tofacitinib 5 mg/12 h
Rituximab	Immunosuppressive agent	1 g given twice within a 2-week interval
IVIG	Immunomodulator	2 g per kg every 4–6 w

*IVIG* Intravenous immunoglobulins; *JAKi* Janus kinase inhibitors; *p.o. per os; w* week

**Table 2:** Summary of recommendations for management of IIM-ILD

Recommendations for management of IIM-ILD: first ILD treatment For people with IIM-ILD we conditionally recommend glucocorticoids as a first-line ILD treatment For people with MII-ILD, we conditionally recommend mycophenolate, azathioprine, rituximab, and cyclophosphamide as first-line ILD treatment options. CNIs and JAKi are conditionally recommended also as a first line treatment. For people with MII-ILD, we conditionally recommend *against* leflunomide, methotrexate, TNFi, and abatacept as first-line ILD treatment For people with IIM-ILD we conditionally recommend *against* nintedanib as a first-line ILD treatment option For people with MII-ILD, we conditionally recommend *against* pirfenidone as a first-line ILD treatment option For people with MII-ILD receiving mycophenolate without evidence of ILD progression, we conditionally recommend *against* adding nintedanib or pirfenidone to mycophenolate For people with IIM-ILD, we conditionally recommend *against* upfront combination of nintedanib or pirfenidone with mycophenolate over mycophenolate alone as first-line ILD treatment options. For people with IIM-ILD, we conditionally recommend JAKi as a first-line ILD treatment option. For people with IIM-ILD, we conditionally recommend against IVIG or plasma exchange as first-line ILD treatment options. For people with IIM-ILD, we conditionally recommend optimal medical management over referral for stem cell or lung transplantation as first-line ILD treatment options Recommendations for management of IIM-ILD progression despite first-line ILD treatment For people with IIM-ILD, we conditionally recommend against using long-term glucocorticoids. For people with IIM-ILD progression despite first ILD treatment, we conditionally recommend mycophenolate, rituximab, cyclophosphamide, and nintedanib as treatment options For people with IIM-ILD progression despite first ILD treatment, we conditionally recommend against adding pirfenidone as a treatment option For people with IIM-ILD we conditionally recommend against nintedanib as a first-line ILD treatment option For people with IIM-ILD, we conditionally recommend against pirfenidone as a first-line ILD treatment option For people with IIM-ILD progression despite first ILD treatment, we conditionally recommend against using tocilizumab as a treatment option. For people with IIM-ILD progression despite first ILD treatments, we conditionally recommend using a CNI as a treatment option For people with IIM-ILD progression despite first ILD treatment, we conditionally recommend using JAKi as a treatment option. For people with IIM-ILD progression despite first ILD treatment, we conditionally recommend adding IVIG as a treatment option. For people with IIM-ILD progression despite first ILD treatment, we conditionally recommend against using plasma exchange as a treatment option. Recommendations for management of IIM with RP-ILD For people with IIM and RP-ILD, we conditionally recommend pulse intravenous methylprednisolone as a first-line RP-ILD treatment. For people with IIM and RP-ILD, we conditionally recommend rituximab, cyclophosphamide, IVIG, mycophenolate, CNI, and JAKi as first-line RP-ILD treatment options. For people with SARD and RP-ILD, we conditionally recommend against methotrexate, leflunomide, azathioprine, TNFi, abatacept, tocilizumab, nintedanib, pirfenidone, and plasma exchange as first-line RP-ILD treatment options. For people with RP-ILD, we conditionally recommend upfront combination therapy (triple therapy for those with confirmed or suspected MDA-5 and double or triple therapy for those without confirmed or suspected MDA-5) over monotherapy as first-line treatment. For people with IIM and RP-ILD, we conditionally recommend against referral for stem cell transplantation over optimal medical management as a first-line RP-ILD treatment. For people with SARD and RP-ILD, we conditionally recommend early referral for lung transplantation over later referral after progression on optimal medical management

Modified from 2023 American college of rheumatology (ACR)/American college of chest physicians (CHEST) guideline for the treatment of interstitial lung disease in people with systemic autoimmune rheumatic diseases [52]. CNI: calcineurin inhibitors. JAKi: JAK inhibitors. IIM-ILD: idiopathic inflammatory myopathy associated interstitial lung disease. IVIG: intravenous immunoglobulins. RP-ILD: rapidly progressive ILD. TNFi: tumor necrosis factor inhibitor

## Data Availability

No datasets were generated or analysed during the current study.
