# Supplementary material for: Treatment Algorithms for Inflammatory Myopathies in Adults: from Guidelines to Clinical Practice
Source: Curr Treatm Opt Rheumatol. Author manuscript; Available in PMC 2026 Feb 21. (PMC12922664; doi:10.1007/s40674-025-00239-5)
Supplement: Supplementary Material [file NIHMS2140126-supplement-Supplementary_Material.docx]

Supplementary table (S1). Immunosuppressive Agents in Inflammatory Myopathies

| Drug | Dose | Indications | Adverse Effects |
| --- | --- | --- | --- |
| Glucocorticoids | Prednisone 0.5–1 mg/kg/day orally. Consider intravenous methylprednisolone pulses 0.5–1 g/day for 3 days in severe cases. | All clinical manifestations | Hypertension, hyperglycemia, hyperlipidemia, osteoporosis, opportunistic infections, cataracts |
| Methotrexate | 15–25 mg/week subcutaneously | Myositis, cutaneous involvement, arthritis | Nausea, vomiting, stomatitis, leukopenia, hepatotoxicity, pulmonary toxicity (acute pneumonitis). Use with caution in ILD |
| Azathioprine | 2–3 mg/kg/day orally  (determine TPMT for adjust of dose) | Myositis, ILD | Gastrointestinal symptoms, myelosuppression, pancreatitis, hepatotoxicity |
| Mycophenolate mofetil | 720 mg every 12 h orally | Myositis, cutaneious involvement, ILD | Gastrointestinal symptoms, myelosuppression, hypertension |
| Tacrolimus | 0.06 mg/kg/day orally | ILD, panniculitis, myositis | Hypertension, renal failure, gastrointestinal symptoms, ischemic heart disease |
| Cyclosporine | 5 mg/kg/day orally | Cutaneous involvement, ILD, myositis | Renal failure, anemia, hypertension |
| Leflunomide | 10–20 mg/day orally | Arthritis | Diarrhea, alopecia, leukopenia |
| Tofacitinib | 5 mg every 12 h orally | Rapidly progressive ILD, refractory cutaneous involvement | Herpes zoster infection, risk of DVT in elderly patients, risk of malignancy |
| Ruxolitinib | 5–15 mg every 12 h | Checkpoint inhibitor–induced myopathy | Cytopenia (anemia, leukopenia, thrombocytopenia), hepatotoxicity, dyslipidemia |
| Cyclophosphamide | i.v. 0.5–1 g/m² monthly or 10–15 mg/kg monthly for 6–12 months | ILD, severe myositis? | Myelosuppression, myeloproliferative disorders, hemorrhagic cystitis, bladder cancer, infertility |
| Intravenous immunoglobulin (IVIG) | 0.4 g/kg/day for 5 days every 4–6 weeks | Myopathy, dysphagia | Hypotension, anaphylaxis, headache, aseptic meningitis, renal toxicity, risk of DVP |
| *Biological Agents* |  |  |  |
| Rituximab | Two doses of 1 g i.v., 14 days apart | Refractory or severe myositis, ILD, and arthritis | Infusion reactions, progressive multifocal leukoencephalopathy |
| Abatacept | 125 mg/week subcutaneously | Arthritis, myocarditis in patients with checkpoint inhibitor–induced myopathy | Upper respiratory tract infections |
| Tocilizumab | 162 mg/week subcutaneously | Arthritis | Upper respiratory tract infections, hypercholesterolemia, leukopenia, neutropenia, hepatotoxicity |

*Abbreviations:* HTN = hypertension; CKD = chronic kidney disease; ILD = interstitial lung disease; DVT = deep vein thrombosis.
